# Modeling and Analysis of Learners' Emotions and Behaviors Based on Online Forum Texts

**DOI:** 10.1155/2022/9696422

**Published:** 2022-01-20

**Authors:** Mingyong Li, Mingyuan Ge, Honggang Zhao, Ziye An

**Affiliations:** College of Computer and Information Science, Chongqing Normal University, Chongqing 401331, China

## Abstract

Under the continuous impact of the epidemic, online learning methods represented by MOOC have developed rapidly. The course forum area has produced a large amount of text-based unstructured data, which can reflect the potential characteristics of learners' emotional states and behavioral interactions, and has an important impact on students' learning outcomes. To this end, this paper constructs an emotional and behavioral analysis model based on online forum texts, obtains forum data from the “Python Language Programming” course on the Chinese University MOOC platform, uses domain dictionary emotion classification method to analyze learning emotions, and based on the method of cognitive behavior coding table and knowledge construction behavior coding table analyzes learners' cognitive behavior and knowledge construction behavior. It can dynamically analyze learners' emotions, behavior changes, and evolutionary trends. This research provides opinions and suggestions on the improvement of platform interactive functions for teachers' online teaching, students' online learning, and platform management, which can effectively improve the efficiency and effectiveness of online learning.

## 1. Introduction

In the context of the deep integration of artificial intelligence and big data in education, significant changes have taken place in the ecological environment of education in China, and the future classroom will no longer be limited to classroom teaching, but to the network, digital, virtualization, and intelligent teaching environment [1, 2]. In the future, classrooms will no longer be limited to classroom teaching but will be networked, digitized, virtualized, and intelligent. Under the influence of the new crown epidemic, online learning methods have developed rapidly. According to statistics from the Ministry of Education, as of May 8, 2020, 1.03 million teachers across the country have opened 1.07 million online courses. Currently, there are 12.26 million online courses, involving various disciplines [3].

As a platform for learners and teachers to exchange and study, the MOOC forum area is an important place to share personal views and release information and resources [4]. However, most learning platforms only provide structured data such as the number of posts, comments, and replies. Such simple quantitative statistics cannot yet provide a more complete assessment of learners' learning effectiveness and learning emotions, etc. It is the unstructured data contained therein that contains certain implicit interactive behaviours and emotional states, but often these unstructured data are rarely used by people [5, 6]. The mining of forum data is not only an important basis for judging the quality of teaching and learning, but also an important indicator of the degree of interaction and communication between teachers and students, and can create a better teaching and learning experience by exploring the interaction process. How to more deeply mine text-based unstructured data, achieve quantitative characterisation of emotional and behavioural data, complete the evaluation of the quality of online learning, and adapt to the diversification of interaction models should be a pressing issue at present [7, 8]. Text analysis of forum interaction areas is significant for teachers to guide virtuous learning behaviours and avoid undesirable ones [9].

The current research on MOOC learning experience is relatively scarce [10, 11]; particularly, the influence of emotion on MOOC learning has not been fully studied [12, 13]. Only by truly understanding the emotional experience of MOOC learners can we reflect on and optimize the course design and implementation in a targeted manner, improve the learning effect of MOOC, and even customize the learning experience of learners.

In summary, this study has the following main contributions:

1. This paper constructs an emotion and behavior analysis model based on MOOC forum text. The model includes two parts: domain dictionary-based sentiment analysis and LSA-based behavior analysis, which dynamically analyzes the characteristics and evolution of learners' emotions and behaviors.
2. Using the emotion and behavior analysis model we proposed, we conducted an empirical analysis on the MOOC course “Python Language Programming.” Studies have shown that learning emotions are dominated by positive emotions, and they show certain evolutionary laws over time. There are also significant differences among different academic achievers; low-level cognitive behaviors and knowledge-building behaviors in learning behaviors account for a relatively large proportion and differ in academic performance. There are also significant differences among achievers.
3. Based on the results of empirical analysis, some operable forum interaction strategies are proposed for educators, learners, and platform managers.

## 2. Related Work

Related work is divided into two categories, namely, MOOC forum text sentiment analysis and behavior analysis, which are described in detail in the following.

### 2.1. MOOC Forum Text Sentiment Analysis

Recent approaches to the analysis of text-based unstructured data fall into the following three main categories: sentiment lexicon based analysis methods, machine-learning based methods, and qualitative analysis methods that require software support. Current researchers usually use content analysis methods to analyze forum data in various dimensions, such as Feldmand et al. constructing themselves' sentiment dictionaries based on existing dictionaries using Bootstrapping and partial manual annotation [14]. In order to explore the law of emotion, learning style, learning activities, and other factors evolving over time, Liu et al. proposed an unsupervised model (TEAM), which explored the different characteristics of emotion changes in the whole learning group and achiever at different levels in depth [15]. Zhang et al. used an existing ontology library lexicon combined with rooting theory to analyze the sentiment of microblogging emergencies and proposed corresponding guidance strategies for public opinion [16]. Wen et al. [17] analyzed the emotions expressed by students in MOOC forum posts by using a dictionary-based emotion classification algorithm and further explored the significant relationship between student learning emotions and the number of students dropping out. However, the classification accuracy of this method mainly depends on the quality of the sentiment dictionary.

In Qin Changbo's study, machine learning and natural language processing methods were used to text-mine classroom discussion posts in Chinese MOOC forum areas, classifying them into three main categories, confusion, explanation, and irrelevance, and built a data mining system for MOOC forum posts [18]. In Hui's research, a fused emotion lexicon and machine learning approach is used to deeply mine the emotional state of the learning experience text [19]. Fei et al. took the MOOC forum text data as the database, combined word2VEc with machine learning algorithm, and obtained the emotional changes and emotional communication of learners, thus improving the learning efficiency [20]. Ling Wang et al. proposed a semantic analysis model (SMA) to detect the emotion of different types of learners' learning states and provide personalized guidance for improving the completion rate of courses [21]. Xu et al. conducted an emotional confrontation study on the interaction data of AlphaGo topics in Zhiwang based on the Senta sentiment analysis model of the Baidu platform and found that, in the AlphaGo topic discussions, “pro-AlphaGo” and “anti-AlphaGo” are significantly confrontational [22]. Shan et al. used Nvivo12 software to qualitatively analyze teacher education catechisms in terms of overall course emotional disposition, course stages, and emotional experience pointers [23].

In recent years, neural network-based sentiment classification methods have been gradually adopted by many researchers. Unlike models that use traditional machine learning algorithms for sentiment classification, neural network-based sentiment classification methods can automatically extract text features. Although there are relatively few emotion classification methods based on neural networks in the MOOC field, this method has been widely used in emotion classification in other fields in NLP [24]. Rao et al. [25] used a two-layer LSTM to predict the sentiment of a document. This method can extract deep semantic information. Tang et al. [26] used a two-way LSTM and attention model to handle sentiment classification tasks. Subsequently, more and more researchers try to use these network model variants and their combinations for text sentiment classification tasks. Huang et al. [27] used a layer of CNN and a double-layer LSTM to jointly capture text features for sentiment classification. Recently, some researchers have proposed a parallel combination of CNN + LSTM network structure and self-attention mechanism for the MOOC course review sentiment classification task, so as to better retain the features extracted from the network [28].

In addition to the above three methods, it also contains studies combining multiple methods, such as Zhu and other researchers who drew on the prefatory idea of affective computing, integrated the use of affective computing, text mining, social network analysis, and multiple regression to establish an affective measure based on an effective weight dictionary for online activity texts, and used regression models to analyze the relevant influencing factors of academic emotions in online activities, providing a harmonious interpersonal environment [29]. There are no absolute advantages or disadvantages of the above-mentioned analysis methods, and data analysis and interpretation is not a simple issue, but also requires the sincere cooperation of other parties such as the platform owner, analysis experts, catechism researchers, course teams, and participating members to provide a comprehensive and professional effort for the forum text study.

### 2.2. MOOC Forum Text Behavior Analysis

The data of learners in the forum area often contains rich semantic information, which maps the behavior patterns and evolution rules related to learning tasks. Moreover, existing studies have shown that as far as learning analysis subjects are concerned, relatively few in China pay attention to students' behaviors [30], while students' subjects are regarded as the research focus in foreign countries [31, 32]. For example, the research group of Carnegie Mellon University in the United States developed behavior codes based on the ICAP framework (Interactive, Constructive, Active, and Passive), and divided the data in the forum area [33]. Wang Jianyuan of the University of Pennsylvania and others used the key discourse word matching method to analyze the cognitive behavior of the MOOC forum area data and summarized the learner's interactive behavior and cognitive rules [34]. There are also some researchers exploring the learning process from the three dimensions of emotion, cognition, and metacognition, and the results show that learning emotion affects cognitive behavior [35].

Wang et al. classified the cognitive participation degree of learners' forum posts according to certain coding manuals, and the results showed that learners with higher-order thinking could gain more knowledge [36]. Therefore, domestic scholars such as Yang at al. not only analyzed user behavior patterns based on behavioral data in the process of collaborative knowledge creation, but also analyzed the sequence transformation of knowledge construction behavior in the process of collaborative translation. They are also the first scholars to explore the implicit interaction behavior in the forum in China [37]. This was followed by Liu and other scholars who developed rules for rating discourse cognition and counted the types of learners' cognitive behaviors based on an automated coding mechanism for semantic content and then used chi-square tests and lagged sequence analysis to verify the differences in key cognitive behaviors and their sequence patterns across different groups [38]. Maldonado-Mahauad et al. used the method of process mining to mine the sequence patterns of learners' interactive behavior and establish a correlation with the strategies in SRL theory [39]. Yue et al. used sequential and clustering algorithms to mine online self-directed learning behaviours and used the learning behaviour patterns of high-achievers as a strategy for intervention experiments with low-achievers, and the experimental results showed good instructional effects [40].

Some researchers have also explored the learning process in terms of three dimensions: emotional, cognitive, and metacognitive, while the results showed that learning emotions affect cognitive behavior [35, 41, 42]. For example, Wang Yun et al., based on the cognitive classification system of Cai Jin and the emotion classification method of Harris et al., used the content analysis method and the lagging sequence analysis method to explore learners' emotions and cognitive behavior patterns and their mutual relationships. The study found that most learners present descriptive, inferring, and explanatory behaviors. The cognitive behavior pattern has hierarchical characteristics; describing behaviors can lead to negative emotions, while comparing, inferring, or explaining behaviors can lead to positive emotions [43]. Similar research methods were also studied by Huang et al. [41]. Hernandez-selles et al. established an empirical global model in their research, which can better predict the relationship between emotions and interactions between teachers and students, and groups in the process of online collaborative learning [44]. The study by Huang Tao et al. argues that in the future intelligent education scenario there should be a tendency to dig deeper into a variety of potential characteristics of learners' knowledge, cognition, emotion, and interaction, in order to deeply explore the intrinsic learning occurrence mechanism of learners' knowledge construction patterns, cognitive development patterns, and emotion occurrence mechanisms, so as to achieve the purpose of multiple and accurate detection of the learning process and learning effects [45]. Wu et al. provided technical and theoretical assistance for the effective fusion of multimodal data from the perspectives of methods and strategies, which in turn provide more possibilities for the mining of educational big data [46]. In addition, the so-called “black boxes” such as MMLA and computer technology have been used in the study of Spikol et al. to observe the multimodal data generated by students in the process of project learning, so as to provide help for effective teaching for teachers [47]. Therefore, this study will comprehensively analyze the intrinsic mechanisms of learner emotions and behaviors in the forum environment and develop effective interaction strategies for relevant users.

In addition to this, Wang et al. conducted a visual analysis of the literature related to international educational text mining in the last decade and found that the current research methods show diversity, with research hotspots focusing on five areas: learning behaviour, cognitive behaviour, behavioural analysis of knowledge construction, writing evaluation, and development of mining tools for curriculum texts [48]. Based on the above research experience, this study uses content analysis and lagged series analysis to analyze the emotions and behaviors of unstructured data in the forum area, so as to comprehensively analyze and deeply explore the intrinsic needs of learners, and provide a basis for improving learners' learning efficiency and MOOC course design.

## 3. Research Design and Methods

**Figure 1:**
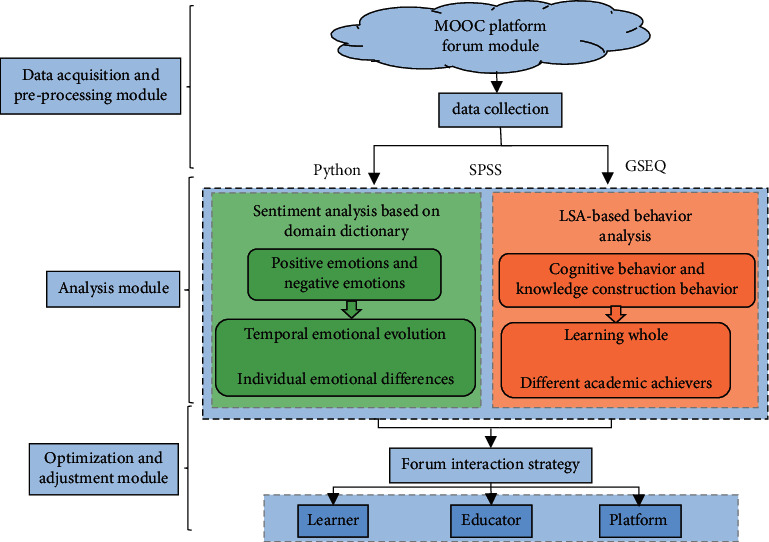
An analysis model of learner emotion and behavior based on MOOC forum text.

The learner's emotion and behavior analysis model based on the MOOC forum text is shown in Figure 1. Among them, the domain dictionary-based sentiment analysis and the LSA-based behavior analysis are the main work of this paper.

### 3.1. Sentiment Calculation Based on Domain Dictionary

In order to perform sentiment analysis on text data, this research uses the domain dictionary method to perform dynamic sentiment analysis on the processed data based on data preprocessing. First, the Dalian Polytechnic Ontology Library dictionary is selected as the basic dictionary, combined with the self-built negative dictionary and the degree adverb dictionary, a domain dictionary suitable for Python programming courses is constructed through the SO-PMI algorithm, and its effectiveness is verified; the accuracy rate is 97.1%, the recall rate is 96.8%, and the *F* value is 97%, which can better identify the emotional tendency of the forum text.

Each forum text may contain both positive and negative emotions. For this reason, this study uses sentiment values to represent forum emotions. The specific calculation process is as follows.

**Step 1 .**

Find the emotional words contained in each forum text. If an emotional word appears in this forum, the word polarity is assigned the corresponding value, the commendatory meaning is assigned −1, and the derogatory meaning is assigned 1. The formula for the sentiment value of a word in this dictionary is

(1) $$\begin{array}{c} z\left(w\right)=i\left(w\right)p\left(w\right). \end{array}$$

Here, *i*(*w*) represents the emotional strength of the vocabulary; *p*(*w*) represents the emotional polarity of the vocabulary; and *z*(*w*) represents the emotional value of the vocabulary.

**Step 2 .**

Find out negative words. If the number of negative words is odd, the polarity of the negative words will be reversed. If it is an even number, the polarity does not change.

**Step 3 .**

Find adverbs of degree. The various degree adverbs are integrated into 6 grades, and different weight values are assigned to them according to the gradient descent formula, as shown in Table 1.

The gradient descent formula used is

(2) $$\begin{array}{c} T_{k+1}=T_{1}{\left(\frac{\sqrt{2}}{2}\right)}^{k},\;k=1,2,3,4,5, \end{array}$$

where the gradient decline rate is $\sqrt{2}/2$ and *T*_1_ is the weight value of the first rank “most.”

**Step 4 .**

Calculate the sentiment value of each forum. The number of negation words and degree adverbs before and after the word and their relative positions were found for each sentiment word in the forum as shown in the following formula.

(3) $$\begin{array}{c} E={\left(-1\right)}^{n}\cdot \left(\sum_{i=0}^{i}h\right)\cdot e\cdot p, \end{array}$$

where *E* represents the sentiment value of a single sentiment word class in each forum; *e* represents the sentiment value of a particular sentiment word; *n* represents the number of negatives associated with the sentiment word; (−1)^*n*^ represents the total number of weights of negatives; *i* represents the number of degree adverbs preceding the sentiment word; ∑_*i*=0_^*i*^*h* represents the cumulative sum of the weights of all degree adverbs preceding the sentiment word; and P represents the relative position of negatives and degree adverbs, with the following two relationships:

(4) $$\begin{array}{c} p=\left\{\begin{array}{c} 0.5,\;\text{negation before an adverb of degree,}\\ 1,\;\text{adverbs of degree before nagatives.} \end{array}\right. \end{array}$$

Each comment contains multiple sentiment words, and the sentiment value of all sentiment words in the text is accumulated to the final sentiment value, calculated as shown in equation (5). In equation (5), *j* represents the total number of sentiment word categories contained in each comment; *Z* (*j*) represents the final sentiment value of each forum, if *Z* (*j*) > 0 means the forum sentiment is positive, *Z* (*j*) = 0 means the forum sentiment is neutral, and *Z* (*j*) < 0 means the forum sentiment is negative.

(5) $$\begin{array}{c} Z_{\left(j\right)}=\sum_{j=1}^{j}E, \end{array}$$

**Step 5 .**

*Domain Lexicon Construction*. This study will use a statistical approach to construct a domain sentiment lexicon, firstly using PMI to count the similarity between two words and then expanding the base lexicon with sentiment words through the SO-PMI algorithm to select extended sentiment words from the forum comments, which in turn increases the domain applicability of the sentiment words. The sentiment value of a word in the forum is shown in the following equation:

(6) $$\begin{array}{c} \text{SO}-\text{PMI}=\left\{\begin{array}{c} >0,\;\text{word has a positive emotional disposition,}\\ =0,\;\text{word has no emotional tendency, it is a neutral emotional word}\\ <0,\;\text{word has a negative emotional orientation.} \end{array}\right., \end{array}$$

Here, word is a word with uncertain sentiment polarity and *P*word and *N*word are positive sentiment seed words and negative sentiment seed words. If the value is greater than zero, then it is a positive sentiment word and if it is less than zero, then it is a negative sentiment word.

### 3.2. LSA-Based Behavioral Analysis

The basic steps of behavioral analysis are (i) to develop behavioral coding rules, (ii) to collect textual data related to behavior, (iii) to code the data one by one according to the format required by the GSEQ software, (iv) to check the consistency of the coding table, (v) to conduct behavioral analysis and obtain the corresponding behavioral conversion frequency table and the adjusted residual table, (vi) to plot the conversion sequence for significant behavior, and (vii) to interpret the questions, the behavioral analysis of which includes two main types of cognitive behavior and knowledge construction behavior. The cognitive behaviour coding table is shown in Table 2, and after two rounds of consistency testing, the final Kapp coefficient was 0.708, indicating strong consistency in the coding results. The coding of knowledge construction behaviours is shown in Table 3, with a final Kapp coefficient of 0.803 after two rounds of consistency testing, indicating a strong consistency of the coding results.

## 4. Experiment and Analysis

In the experimental part, our research object is the course of “Python Language Programming.” On the basis of data collection and preprocessing, we focus on the analysis of learners' emotion and behavior and get some rules. The details are described as follows.

### 4.1. Study Subjects

In this paper, we selected a representative course of the Python category with a high number of participants and a high overall rating among Chinese university mooted courses: the course “Python Language Programming.” Considering that the eleventh course (February 18, 2020, to May 12, 2020) is the longest of all the courses offered, and this period is a critical stage in the prevention and control of the new crown epidemic, the data from the eleventh course is used as the data source for this study. With over 760,000 learners participating in this course, and in the home independent learning phase, this period has a higher number of participants and forum interaction data compared to other open periods.

### 4.2. Data Acquisition and Preprocessing

Firstly, a total of 17,814 threaded posts and replies were obtained from the course forum area through web crawling. Secondly, the raw data was preprocessed by de-duplication and deactivation of words. Then, all the processed data were sorted according to the number of weeks and days, which were divided into 0 to 11 weeks, a total of 12 weeks of data, and saved as 0z.xlxs-11z.xlxs, and then divided into days and all the periods of each day on a weekly basis, and saved as 2.22Saturday.xlxs. Finally, in order to analyze learners' emotions and behaviors in a fine-grained way from different dimensions, it is necessary to classify learners who have obtained excellent certificates as high academic achievers and learners who have obtained passes as medium academic achievers, and then to organize the postings of different academic achievers in chronological order.

The content of the postings of the different academic achievers is then organized in chronological order. The analysis and interpretation of learning emotions and behaviors is carried out at a fine-grained level at both the overall and individual levels. On the one hand, the analysis helps teachers to observe the overall attitude of students towards the forum, and on the other hand, the individual level analysis helps teachers to detect abnormalities from the perspective of the average level and to provide timely attention and intervention.

### 4.3. Learner Emotion Analysis

Next, it mainly analyzes and interprets learners' emotions from two levels of the whole and the individual: on the one hand, according to the order of time evolution, the study of the overall emotion transfer law and on the other hand, from the individual to explore the emotional differences between different individuals and different academic achievements.

#### 4.3.1. Temporal Emotional Evolution

This paper analyzes the learning emotions of online learners in terms of temporal evolution, including classified statistics, visual display, and dynamic analysis. It mainly includes two types: one is the academic emotion distributed throughout the entire learning process, also known as the duration academic emotion; the other is the real-time academic emotion distributed in different time stages (such as weekly and daily time intervals).

*(1) Periodic Sentiment Analysis*. The statistics yielded a total of 57% of the total forum posts for positive emotions and 43% of the total discussion posts for negative emotions. It was observed that learners' overall evaluation of the course was relatively stable, with a high proportion of positive sentiment being expressed. The results indicate that learners generally expressed appreciation and satisfaction with the Python course, but also expressed some negative sentiment towards the course.

The following reasons were identified after analyzing the current situation: as a programming course, Python programming requires learners not only to learn theoretical basics but also to write code themselves and practise their practical skills [49]. For example, in the second week of Python basic graphics and the case study in this chapter, students developed their creative thinking through hands-on programming and active thinking. The development of thinking increases students' interest and motivation in learning. Unlike the Arts course, the weekly passing tests in the Programming course are presented in the form of game-based cases that synthesise students' prior knowledge and make the post-course work interesting. However, there is also a certain percentage of negative emotion in the overall learning process, so a certain threshold of negative emotion can be set in the actual online course, and if the learning emotion exceeds the specified threshold, a corresponding warning message will be sent to the teacher or course manager, which will help the teacher to grasp the dynamic emotional state of the students more accurately and intervene in time to help students adjust their learning state.

**Figure 2:**
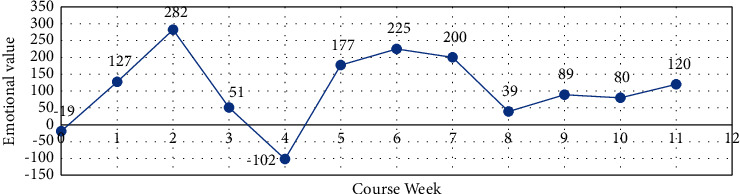
General evolutionary trends in emotional disposition.

**Figure 3:**
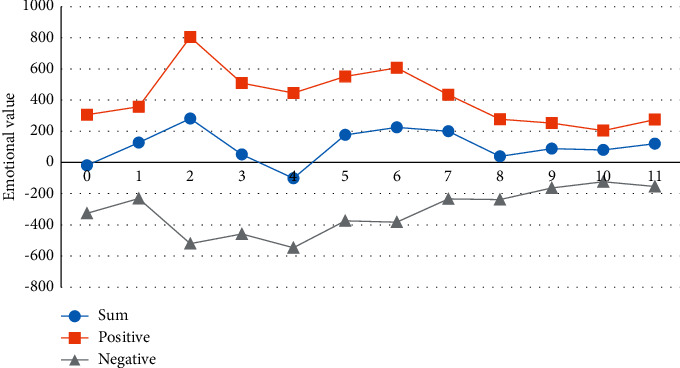
General evolutionary trends in learners' different emotions.

*(2) Weekly Sentiment Analysis*. As learning emotions are dynamic, the interval between observations of learning emotions should not be too long (e.g., one month), as the details of emotions may be missed if the interval is too long; however, the interval should not be too short (e.g., one hour), as the observed changes in emotions may be unstable if the interval is too short. Taking these factors into account and taking into account the teaching schedule, a week's span (from 0:00 on Monday to 24:00 on Sunday) is considered to be a more stable and observable period of time and is used as a time cycle. Figures 2 and 3 plot the trends in the evolution of emotions over the twelve course weeks, and it is observed that the trends in the evolution of positive and negative emotions are generally consistent, and the trends in the evolution of positive and negative emotions are also generally consistent.

In this study, the whole learning process was divided into three stages: the early learning period (week zero to week two), the middle learning period (week three to week seven), and the end learning period (week eight to week eleven), and the distribution of learning emotions throughout the course week was observed. There was a consistent trend of positive and overall mood swings.

The trend of the learners' emotions in the eleventh course was as follows: as the students had just returned from their holidays at the beginning of the course, they were in the stage of exploring the online learning method, so their overall learning state was not very good and there was a certain degree of confusion, so their positive emotions were not very high. However, with the adjustment of the state and the continuous exploration of learning methods, the learning mood reaches its peak in the mid-term, which is mainly because the learners have a certain enthusiasm and interest in Python learning at this stage. The learning mood peaks in the middle stages, mainly because there is a degree of motivation and interest in learning Python. However, as the course continues, students accumulate difficulties and problems to a certain extent and dissatisfaction with learning Python exists, and negative emotions peak at this point. Through processes such as teacher guidance and peer support, students are helped to get through the difficult learning period. Towards the end of the school year, near the final exam, there are no significant ups and downs in learning emotions, but problems such as excessive revision and exam pressure are at their lowest point, so it is also important to reduce students' stress in the classroom to some extent.

**Figure 4:**
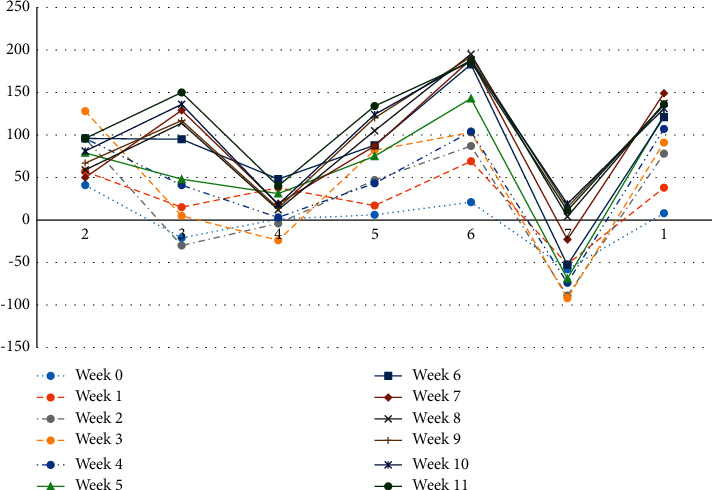
The overall trend in the evolution of sentiment over the days.

*(3) Sentiment Analysis of Days*. To analyze the trends in learners' dynamic mood changes at a more granular level, this study will observe the trends in the evolution of mood dynamics within the weekly subsessions (as shown in Figure 4), considering Tuesday of each week as the start date of each week when the new resources for the course are open. In Figure 4, the horizontal coordinates indicate the seven days of the week, the vertical coordinates indicate the sentiment values corresponding to each day, and the different lines indicate the different times of the week, which overall represent the daily sentiment trends over the twelve weeks.

Looking at Figure 4, we can see that there is not yet a clear consistency in the emotional evolution in the first few weeks, but in the middle and later stages of learning there is a clear consistency in the emotions, indicating that learners can manage their own learning time in the middle and later stages. In terms of the number of weeks that showed consistency, Saturday's learning mood was at its highest point, Sunday's learning was at its lowest mood point of the week, and a low point in learning mood occurred every Thursday. This is probably because most online learners are in a study or work situation and therefore usually choose to take courses on Saturdays and participate more in the forums, where positive emotions are at their peak. Correspondingly, learners do not usually choose to study on Sundays and may mostly choose to take a break on Sundays, so there are less forum interaction and more negative emotions. However, it may be that, to catch up on their learning, they will catch up on the updated content before the next week's course resources open (i.e., every Monday), so that learning sentiment rises significantly on Mondays but then falls again on Tuesdays, rises again on Wednesdays, and then rises again on Fridays. Considering the intense division of time between the days, the mood oscillates throughout the week, but is consistent in the middle and end of the study period, with the highest mood on Saturday and the lowest on Sunday.

#### 4.3.2. Individual Mood Differences

In order to further analyze the differences in academic emotions between academic achievers, this study first screened out those individuals who received certificates but did not post comments or posted forum content about coursework, content related to the Python123 platform, and pure code, resulting in 36 individuals in the higher academic achievement group and 9 individuals in the middle academic achievement group. Based on the above groupings, the domain dictionary was used to calculate the corresponding emotion values for each individual, and then an independent samples *t*-test was used to investigate whether there were differences in positive and negative emotions between the different academic achievers.

The results of this analysis showed that there were significant differences in both negative emotions (*p* < 0.05) and positive emotions (*p* ≤ 0.001) between the academic achievers. In terms of means, the high-achieving learners were more active in positive emotions (13.364 > 4.10) than the middle-achieving group, while the middle-achieving group was more active in negative emotions (−19.51 > −7.57) than the high-achieving group, thus considering a correlation between academic achievement and academic emotions. To verify this relationship, the Pearson correlation coefficient in SPSS software was used for analysis. From the results, it is clear that the value of the correlation coefficient between academic achievement and positive emotions is 0.361, indicating a general positive correlation between academic achievement and positive emotions (0.361 < 0.4). The correlation coefficient between academic achievement and negative emotions was 0.539, indicating that there was also a significant positive correlation between academic achievement and negative emotions (*p* ≤ 0.001), and the correlation coefficient of 0.539 > 0.4 indicates that there is a strong positive correlation between academic achievement and negative emotions; i.e., the more the negative emotions, the lower the academic achievement. Based on this result, it is known that negative emotions are more likely to affect the final academic achievement; therefore, it is more important for instructors to pay attention to the abnormal individuals with poor emotions.

### 4.4. Learner Behavior Analysis

This section will provide a fine-grained analysis of learners' cognitive and knowledge-constructing behaviours in different dimensions.

#### 4.4.1. Analysis of Cognitive Behavior of Learners in Different Dimensions

This subsection will provide a detailed interpretation of learner cognitive behaviour along two different dimensions: overall and for different academic achievers.

*(1) Analysis of Overall Cognitive Behavior of Learners*. In this study, the proportion of each cognitive behavior in the whole group was calculated and it was found that descriptive behaviors (43.9%) and inferential or explanatory behaviors (30.1%) were more predominant in the group. Firstly, learners preferred to describe the problems they encountered in their studies or simply state their personal opinions, so descriptive behaviors accounted for nearly half of the group, indicating that learners only thought about and explored the problems on the surface, but did not go deeper to reveal the patterns behind the problems. Also, some learners responded or commented with simple affirmative or negative statements such as “yes, what else do you want,” lacking deeper interaction. Secondly, learners also tend to use inferential or explanatory behaviors to engage in interactive discussions in the forum and also to help other learners solve problems in this way. Then, learners also used a small number of conceptual or definitional behaviors to validate their ideas. Finally, fewer learners used difference-contrast relations to argue the differences that existed between perspectives and were not very keen to explicitly point out points of cognitive difference with the object of their interaction, choosing instead to avoid talking about them. Comparing several types of cognitive behavior reveals that comparative cognitive behavior is the least frequent, but this type of questioning is often the most effective in triggering deeper exploration on both sides of the interaction and in providing a pavement for other participants to refine new ideas, making comparative cognitive behavior an important part of higher-order thinking for learners. In conclusion, the high percentage of low-level cognitive behaviors (69.9%) and the lack of cognitive behaviors that enhance higher-order thinking in the interactive communication process suggest that guidance from teachers or other instructional managers such as teaching assistants is urgently needed in the learning process.

*(2) Analysis of Cognitive Behavior of Learners with Different Academic Achievements*. The willingness to learn, motivation, and learning goals of different academic achievers may differ, giving rise to differences in interactive behaviors. To explore the differences in learner cognitive behaviors among different academic achievers, it was necessary to eliminate the non-cognitive behavior postings and finally screen out 36 higher academic achievers and 9 middle academic achievers. The coded cognitive behavior data were subjected to chi-square testing and the results showed that there were significant differences in these four cognitive behaviors between the academic achievers (*p* ≤ 0.001).

Higher academic achievers had the highest proportion of inferred or explained high-level cognitive behaviors (62.67%) and middle academic achievers had the highest proportion of described low-level cognitive behaviors (47.37%), indicating that higher academic achievers had higher levels of cognitive behaviors. The reason for this may be due to the difference in the total number of posts between higher academic achievers and middle academic achievers and the fact that a large number of posts without cognitive behaviors were removed from the posts of middle academic achievers. The reason for this phenomenon is most likely that these learners just want to post more content such as punch cards, flooding posts, and logistical posts to complete the requirements for the completion certificate. Comparing the descriptive behavior posts, the proportion of descriptive behavior posts made by moderate achievers was about three times higher than the proportion made by higher achievers, suggesting that moderate achievers are more willing to describe facts or phenomena. In comparing the inferential or explanatory behavior posts, the proportion of higher academic achievers was about twice as high as the proportion of intermediate achievers, suggesting that higher academic achievers are more likely to actively think about the content, explore the principles behind the knowledge, or give examples of cause-and-effect relationships between events and propose new solutions and ideas. The above analysis shows that the higher achievers tend to post definitions, inferences, or explanations, and to think in terms of definitions and continuous thinking; the middle achievers tend to post descriptions, inferences, or explanations, and to think in terms of asking a lot of questions and trying to solve problems. Teachers are also called upon to help moderate achievers build their own cognitive strategies to facilitate meaningful learning.

#### 4.4.2. Analysis of Learners' Knowledge Construction Behavior in Different Dimensions

This subsection will provide a detailed interpretation of learners' knowledge construction behaviour along two different dimensions: overall and for different academic achievers.

*(1) Analysis of the Overall Knowledge Construction Behavior of Learners*. Firstly, the knowledge construction behavior of the whole group was measured, and it was found that 40.6% of the interactive posts were opinion sharing posts and 36.5% were meaning construction posts, which were the two largest sections, and the total ratio was 77.1%, indicating that most of the learners were in the low-level construction stage of opinion sharing and meaning negotiation. The results suggest that learners tend to simply state their opinions or ask questions about code bugs or software installation and then elaborate on the details of their questions and discuss with others the possible causes of the problems, but are not sure of the true causes of the bugs and do not agree on them over the interactions. Secondly, the proportion of questioning posts was 2.8%, indicating that only some of the learners tested their own coding process and found that the answer did not match the reference answer, expressed that the final result of their code was different from the expected result, or expressed a different view from the questioner, or even denied the other person's view directly. The reason for this may be that learners are only at the stage of acquiring knowledge in online courses, but not at the stage of thinking and reflecting on their learning. There is no doubt that the ability to question is an important learning skill, and with so many online learning resources, learners are required to learn to reflect and question rather than read a dead book. Teachers are also asked to pay more attention to the questioning posts posted by learners to help students practise their thinking skills. Finally, it was found that the proportion of test or revision posts was 17.5% and the proportion of application posts was 2.6%, both of which were relatively low overall and at a high level of construction. This suggests that it may take time and effort for learners to complete the acquisition of knowledge and move up to the revision and application stages, resulting in fewer high-level knowledge construction behaviors. Higher level knowledge construction requires a degree of creativity and innovation and is a difficult task, but it may also be due to a lack of critical thinking on the part of the learner to find the right way to correct the problem [50, 51]. Learners are therefore required to exercise higher-order thinking in online learning.

**Figure 5:**
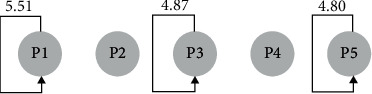
Learning overall knowledge construction behavioral transformation diagram.

The sequential transformation of knowledge construction behavior of the learning as a whole is plotted according to the residual table, as shown in Figure 5. It can be seen from the figure that, in the learning process, learning tends to repeat a certain behavior, such as constantly asking questions (P1 ⟶ P1), negotiating meaning (P3 ⟶ P3), and applying (P5 ⟶ P5). The overall behavior may involve the learner asking a question and then the interactor giving his or her code for reference; it may also occur that when the learner asks a question, the two interactors discuss and negotiate with each other, or of course that when the learner finds the cause of the problem, the other interactor returns to the concept or theory itself to explain it in detail, and some interactors are able to give examples in relation to the theory and clearly identify the cause of the error.

(2) *Analysis of Knowledge Construction Behavior of Learners with Different Academic Achievements*. The differences in willingness to learn, motivation, and goals of the academic achievers may lead to slight differences in knowledge construction behaviors. In order to ensure the accuracy of the differences in knowledge construction behaviors between academic achievers, posters without knowledge construction behaviors were eliminated in advance, and the knowledge construction behaviors of 36 higher academic achievers and 9 middle academic achievers were finally obtained after screening, and the cardinality test was conducted to verify the level of differences, and the results showed that there were significant differences between academic achievers in these five categories (*p* = 0.003 < 0.05).

Analysis of the proportion of posts for each behavior revealed that higher academic achievers posted the largest proportion of shared views posts and middle academic achievers posted the largest proportion of negotiation of meaning posts. The analysis revealed that the higher achievers were more willing to ask questions or share their opinions and that they were not just passive learners, but also active thinkers. Therefore, teachers should encourage learners to ask more questions to help them solve their own problems while practising their own logical expression. The intermediate achievers are more willing to interact with their peers, indicating that they are more willing to participate in the search for solutions to problems, possibly because they have encountered similar problems and are trying to find solutions through meaningful negotiation, but also as a warning to teachers that they should provide more guidance on learning methods. Comparing the levels of knowledge construction between high and middle academic achievers revealed that higher academic achievers had a greater proportion of higher knowledge construction behaviors (14.76% > 7.50%), indicating that higher academic achievers had a somewhat higher level of knowledge construction.

**Figure 6:**
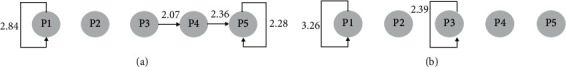
Sequence of knowledge construction behavioral transitions among academic achievers.

The knowledge construction behaviors of the different academic achievers were analyzed to obtain a sequence of significant behaviors transformation charts, which yielded the results shown in Figure 6. The more significant behavioral sequences of the higher academic achievers are P1 ⟶ P1, P5 ⟶ P5, and P3 ⟶ P4 ⟶ P5, indicating that the higher academic achievers are good at thinking and asking questions in a continuous cycle and can interpret the meaning of the concept or term itself according to their own understanding of the metacognitive level of knowledge. In addition to this, the higher achievers have shifted from lower to higher levels of knowledge construction; for example, after negotiating meaning with peers, they have tested and corrected their own errors, have increased their metacognitive level, and have changed their knowledge structures and ways of thinking. The more significant behavioral sequences for the moderate achievers were P1 ⟶ P1 and P3 ⟶ P3. The comparison revealed that the behavioral sequences for the moderate achievers were more homogeneous and were dominated by the repetition of certain behaviors. For example, when learners share their ideas, some learners simply give their own code or website for reference during the interaction; some learners directly tell each other that the teacher has explained this knowledge in the lesson; and some learners agree with the ideas upstairs and discuss and negotiate with each other to a certain extent, which may be due to the fact that the intermediate achievers simply want to obtain a certificate.

Comparing the sequence of cognitive behavioral transitions of the different academic achievers, it can be seen that the secondary academic achievers lack a sequence of behavioral transitions from low-level knowledge construction to higher-level knowledge construction, such as testing and revision after meaning construction (P3 ⟶ P4) and application after testing and revision (P4 ⟶ P5), and the lack of development of these behavioral levels also confirms that the transition from the secondary academic achievers to the higher academic achievers requires teachers' support. The lack of development at these levels also confirms the need for teachers to provide optimal training to help them reach a deeper level of knowledge construction as they move from middle to high academic achievement.

## 5. Discussion and Recommendations

In order to inform both teachers and learners, this study takes into account the current state of forum interaction in the Python programming course and proposes several actionable forum interaction strategies for teachers, learners, and platform administrators.

### 5.1. Strategies for Pedagogues

The pedagogue should be personally engaging, appropriately paced to enhance knowledge, improve data literacy, provide timely feedback and take a leading role, and provide personalized support services on this basis.

#### 5.1.1. Enhancing the Personal Appeal of the Teacher

In the course of the data analysis, it was found that learners expressed a lot of admiration and praise for the course instructor, which shows that the instructor's teaching methods, presentation format, personal charm, and other teaching behaviors have an important impact on the construction of knowledge and deep interaction with students.

#### 5.1.2. Rationalising Teaching Pace and Enhancing Intellectual Interest

In the design of the lesson, for example, the part on “Python Basic Image Drawing,” the practicality and interest of the content should be enhanced in order to stimulate students' interest in learning. In addition to this, teachers should also control the difficulty of teaching according to the level of difficulty; for example, week 0 and week 1 should increase the teaching pace on the basis of the original teaching schedule, but should also give students a buffer time to enter the learning state. After the second week, which is a critical stage of learning, you need to start paying attention to the learning mood of each student and slow down the learning pace appropriately. However, after the fourth and fifth weeks of difficult content, the pace of learning needs to be slowed down and the focus should be on the quality of teaching.

#### 5.1.3. Improving Data Literacy

As there is a lot of implicit data related to learning on the platform, in order to read and understand the data, you need to take initiative and learn the core techniques related to the application and practice of big data in education, data processing and analysis, analysis and interpretation of data results, etc., through various means. Consciously develop a sense of data awareness and adjust teaching strategies in depth through deeper digging for the purpose of optimising teaching effectiveness.

#### 5.1.4. Providing Timely Feedback

Teachers or teaching assistants should give full play to their leading role, posting more open questions in the forum, scaffolding critical questions for students, encouraging students to put forward different views, promoting students' knowledge construction, and developing higher-order thinking skills. In addition to this, teaching assistants should take the lead and help the forum to develop in the right direction. When lecturers post topic posts in the forum, they should avoid leaving comments under the topic posts to suggest general ideas for students, and encourage more postings, different views, and the courage to question them to facilitate the construction of meaning by other learners [52]. In addition to this, teachers should also guide students to use cognitive tools (such as mind maps or concept maps, etc.) and Python basic image drawing to sort out the views, summarize and organize the different views of the group, to promote the sublimation of views and the level of knowledge construction.

#### 5.1.5. Taking a Leading Role

Take a leading role in observing the interaction in the forum area and use the spare time to respond to simple questions. Posting supportive or favourable comments to meaningful responses encourages the construction of meaningful knowledge and promotes mutual help among students. Consider using live video streaming to answer questions online for learners when they encounter more difficult questions that most students encounter, and keep an eye on the forum interaction strategy.

#### 5.1.6. Providing Personalized Support Services

As a result of the above emotional analysis, teachers should pay particular attention to learners with negative emotions and provide a degree of intervention and guidance to help students get out of their learning difficulties and optimize the learning process and learning outcomes. For example, when negative emotions are high, teachers need to pay close attention to the individual learner, providing emotional cues, teacher guidance, and peer support, such as human-computer, teacher-student, or student-student interaction measures to reduce students' resistance to and fear of difficult problems.

This can be done by considering different teaching modes for different groups of learners, differentiating the course design, and improving the accuracy of online learning, in order to tailor the teaching to the student's needs and achieve precise teaching. In addition to this, personalized emotional profiles can be introduced to facilitate adaptive adjustments in subsequent courses according to emotional dynamics, such as appropriate teaching resources push, so as to improve the positive emotional experience of students, so that their learning interest and initiative to achieve the best [53].

### 5.2. Strategies for Learners

Learners should prepare for lessons, improve their self-regulatory skills, identify cognitive strategies, and exercise higher-order thinking.

#### 5.2.1. Preparing for Classes and Improving Self-Regulation

It takes one to two weeks to get into the swing of things before the course starts, and within this time to prepare for the learning. Firstly, you need to have a general understanding of the course content, and only if you are familiar with the course content can you plan your subsequent time well. Secondly, you need to familiarise yourself with the various functions of the platform and the installation and use of its associated software. Finally, you need to be clear about your motivation for learning, whether it is to obtain a certificate or to acquire knowledge or skills. One particular point to note is that online learning requires not only a sense of self-awareness and active learning but also a certain level of self-learning ability. This self-learning ability requires self-monitoring, which requires learners to plan their time according to the actual situation, such as planning their learning activities, supervising the learning process appropriately, and evaluating the learning results in stages and summaries, on the premise of being active and proactive. The students are active and proactive and plan their time well according to the actual situation, such as planning their learning activities rationally, supervising the learning process appropriately, and doing stage and summative evaluations of the learning results.

#### 5.2.2. Identifying Cognitive Strategies and Exercising Higher-Order Thinking

From the above analysis, it is clear that most learners have not yet reached the desired cognitive depth, so they need to be able to take full advantage of their initiative and have the ability to construct their knowledge, to discover the mechanisms by which knowledge occurs and to explore the laws of its evolution, to achieve knowledge-driven deep learning, and to promote meaningful learning, in addition to learning to analyze, compare, and evaluate issues from multiple perspectives, to use higher-order thinking to construct high-level knowledge, and to transform what is learned into a complete body of knowledge. During forum discussions, it is important to exercise a sense of self-reflection, not to follow the crowd, but to acquire the right learning strategies and then learn to solve difficult problems independently. You should write more hands-on operational programs when studying programming courses in order to exercise higher-order thinking such as problem-based critical thinking and creative thinking.

### 5.3. Strategies for Platform Managers

Platform administrators should categorise the content of forum posts for display, support multiple expressions, visualise implicit behaviours, and focus on course evaluation.

#### 5.3.1. Support for Multiple Expressions

This learning platform currently only supports the posting of text and images, but as Python language programming is an operational course, it is difficult for students to post static forum content such as text and images to fully and accurately express the specific problems they encounter in programming, so the platform could be developed to support the transmission of dynamic content such as video or voice so that both parties can accurately express and understand each other's content. To facilitate deeper insight and analysis for both pedagogues and learners, visualisation can be considered, which can help them to dynamically identify those with learning difficulties and provide timely assistance.

#### 5.3.2. Category Display Function for Forum Posts

To address the current situation of learners posting repetitive content and simple support or approval content in order to obtain certificates, platform administrators could consider categorising the content of posts in the forum area in order to stimulate learners' higher level of knowledge construction and cognition, help students engage in high-quality interactions, and improve the level of interaction. For example, post content could be categorised into top posts (questions posted by teachers), highlight posts (thought-provoking topics, high-quality discussion questions), repeat posts (the same content posted by learners), and stepped posts (possible class swiping posts).

#### 5.3.3. Focusing on Course Evaluation

Observation of the courses on the current Chinese university catechism platform revealed that although the number of participants in the courses was high, the number of people who rated the courses was low and the evaluation dimension was single. In order to obtain learners' perspectives on the course and improve and enhance subsequent courses, platform managers should create a multidimensional and exclusive evaluation scale for this course only and encourage learners to systematically evaluate different aspects of the online course. Course evaluations not only provide timely feedback and suggestions for students and teachers, but also help in the design and development of online courses.

#### 5.3.4. Visualisation of Implicit Behaviour

To facilitate deeper insight and analysis by teachers and students, visualisation can also be considered, for example, by setting thresholds for learner emotions, with the system automatically generating warning signals to teachers or students if a certain threshold is exceeded. The visual displays explore the cognitive and knowledge construction processes of individuals or different groups and adjust discussion activities and interaction strategies. For learners, they can view their personal knowledge based on the platform's intelligent learning analytics mapping and dynamically adjust learning styles.

## 6. Conclusion

The interactive forum area of an online course is an important place for course communication and discussion, and the text data generated in it contains a large amount of implicit information that can be used to explore the emotional state and behavioural characteristics of learners and provide important data support for relevant stakeholders. This study uses the domain dictionary emotion classification method to analyze the emotions of learning, and the cognitive behaviour and knowledge construction behaviour of learners based on the cognitive behaviour coding table and the knowledge construction behaviour coding table, so as to provide reasonable suggestions and opinions for the three major user groups, namely, learners, teachers, and administrators. In future research, the improvement of the model (for example, the use of deep learning methods) is one aspect, and another important aspect is to apply the proposed model to actual teaching scenarios and integrate emotion and cognitive analysis into intelligent teaching systems. The system can provide personalized intervention to online learners, and it can also help teachers grasp the learning status of students in real time and automatically provide online learners with personalized support services to make up for the limitations of “emotional lack” in the intelligent age.

## Figures and Tables

**Table 1:** Dictionary of adverbs of degree.

Grade	Weights	Adverbs of degree (for example)	Quantity
Most	3	Very, extremely, extremely, extreme	69
Super	2.1	Much, floating, too much, excessive	30
Very	1.5	Good, very, good, old, good	42
More	1.06	More, also more	37
Slightly	0.75	Also, strange, some, quite, slightly	29
Less	0.53	A little, not much, not a little	12

**Table 2:** Cognitive behavioral coding table.

Cognitive behavior	Code	Description
Definition	K	Provide definitions of concepts and terms
Description	D	Describe facts or a phenomenon
Compare	C	Provide a description of the relationship between things, subjects, or methods
Extrapolate or explain	E	Describe what happens under certain conditions or give examples to demonstrate the causal relationship between two facts or events

**Table 3:** Coding table for knowledge construction behaviors.

Code	Cognitive behavior	Description
P1	Share your views	Provide definitions of concepts and terms
P2	Raising a challenge	Describe facts or a phenomenon
P3	Meaningful consultation	Provide a description of the relationship between things, subjects, or methods
P4	Testing or revising	Describe what happens under certain conditions or give examples to demonstrate the causal relationship between two facts or events

## Data Availability

The data can be obtained from the corresponding author upon request.
